# Risk Factors for Lateral Margin Positivity Following Endoscopic Submucosal Dissection in Patients with Early Gastric Cancer

**DOI:** 10.3390/cancers18050801

**Published:** 2026-03-01

**Authors:** Min-Kyung Yeo, Sun Hyung Kang, Hyuk Soo Eun, Eaum Seok Lee, Hee Seok Moon, Seok Hyun Kim, Jae Kyu Sung, Byung Seok Lee

**Affiliations:** 1 Department of Pathology, Chungnam National University College of Medicine, Daejeon 35015, Republic of Korea; 2 Division of Gastroenterology and Hepatology, Department of Internal Medicine, Chungnam National University College of Medicine, Daejeon 35015, Republic of Korea

**Keywords:** early gastric cancer, endoscopic submucosal dissection, lateral margin positivity, subepithelial spread, risk factors

## Abstract

Background: Lateral margin positivity (LM+) after endoscopic submucosal dissection (ESD) for early gastric cancer (EGC) may lead to residual disease and additional treatment. Subepithelial (SE) spread beneath normal mucosa has been suggested as a mechanism, but its association with LM+ is unclear. Methods: We retrospectively reviewed patients who underwent ESD for EGC from 2011 to 2021. Twenty-one LM+ cases were identified, and 227 LM– controls treated in 2019 were selected. Clinicopathologic and endoscopic factors were compared, and multivariate logistic regression was used to identify predictors. SE spread extent was evaluated pathologically. Results: Differentiation, Lauren classification, lesion size, and endoscopic color change were associated with LM+ in univariate analysis. Lesion size ≥ 2 cm was the only independent predictor. SE spread length was greater in LM+ cases than controls (5.80 ± 1.30 mm vs. 2.60 ± 2.36 mm, *p* = 0.004). SE spread ≥ 5 mm significantly increased the risk of LM+ (OR 15.077, 95% CI 1.550–146.670). Conclusions: SE spread—particularly when ≥5 mm—may contribute to LM+ by obscuring the true tumor boundary. Wider marking and resection margins may be considered in lesions with features suggesting SE spread.

## Simple Summary

Endoscopic submucosal dissection is a standard treatment for early gastric cancer, but lateral margin positivity remains a clinically important issue because it may lead to residual disease and additional treatment. One proposed mechanism is subepithelial spread of cancer cells beneath normal-appearing mucosa, which can obscure the true tumor boundary during endoscopic resection. In this study, we evaluated clinicopathologic and endoscopic factors associated with lateral margin positivity and focused on the extent of subepithelial spread. Although the mere presence of subepithelial spread was not significantly associated with lateral margin positivity, a greater extent of spread was strongly correlated with positive lateral margins. In particular, subepithelial spread measuring five millimeters or more was associated with a markedly increased likelihood of lateral margin positivity. These findings suggest that lateral margin positivity cannot be explained solely by technical or operator-related factors and that intrinsic tumor growth patterns play an important role. Awareness of extensive subepithelial spread may help endoscopists consider wider resection margins in selected lesions and potentially reduce incomplete lateral resection during endoscopic treatment of early gastric cancer.

## 1. Introduction

Endoscopic submucosal dissection (ESD) is the standard treatment for early gastric cancer (EGC) without lymph node metastasis, offering outcomes comparable to those of surgical resection. However, additional surgical resection may be required if post-ESD pathologic evaluation reveals incomplete resection. Incomplete resection includes cases in which (1) the lesion is macroscopically removed, but the risk of lymph node metastasis remains (e.g., deep submucosal invasion or lymphovascular invasion [LVI]), indicating a beyond-indication case; or (2) postprocedural pathologic examination reveals positive resection margins, with cancer cells identified at the lateral or deep margins.

A study conducted in Japan reported that 55% and 60% of positive lateral and vertical margins, respectively, were attributed to operator misdiagnosis [1]. However, whether such misdiagnoses are solely due to the endoscopist’s insufficient skill or attentiveness remains unclear. A Korean study suggested that a substantial proportion of lateral margin (LM) positivity resulted from incorrect delineation of the resection line during ESD; however, that study did not evaluate the impact of operator experience or interoperator variability [2]. Conversely, a Japanese study proposed three additional causes of LM positivity: laterally spreading flat lesions, the presence of unexpected adjacent lesions, and subepithelial (SE) extension of cancer cells beneath normal-appearing mucosa [3].

The first two factors may be overcome, to some extent, with increased endoscopic experience and improved image resolution. However, for SE extension of cancer cells beneath normal-appearing mucosa, no specific countermeasures exist other than setting a wider resection margin. SE spread of EGC is more common than expected and may occur across all histological subtypes [4]. However, the relationship between SE spread and LM positivity following ESD remains unclear.

This study investigated factors that may contribute to LM positivity after ESD, focusing on the role of SE spread, to provide evidence to help optimize resection strategies and reduce unnecessary additional interventions.

## 2. Materials and Methods

### 2.1. Study Population

We included patients who underwent ESD for EGC at a tertiary referral hospital in Daejeon, Korea, between 1 January 2011, and 31 December 2021, and had pathologically confirmed LM positivity. The control group comprised patients who underwent ESD for EGC in 2019 and were confirmed to have LM negativity. A case–control design was adopted for this study. Because lateral margin positivity after ESD is a relatively rare event, patients with LM positivity were accumulated over multiple years to secure an adequate sample size for analysis. In contrast, using all LM-negative cases from the entire study period as controls would have resulted in a large and heterogeneous cohort, making detailed and consistent endoscopic image review and pathological reassessment impractical. Therefore, a single calendar year was selected for the control group to allow uniform evaluation under consistent endoscopic equipment, procedural protocols, and pathological processing conditions. We excluded patients who underwent piecemeal resection because the resection margin could not be evaluated.

### 2.2. ESD Procedure

ESD was performed by five endoscopists, each with experience performing >5000 upper endoscopic examinations and >200 ESD procedures. All procedures, including ESD and preoperative endoscopic evaluations, were performed using video endoscopy (H260 and H290; Olympus Medical Systems Tokyo, Japan). Indigo carmine chromoendoscopy was performed in all cases.

### 2.3. Pathologic Evaluation

Pathologic specimens obtained by endoscopic submucosal dissection were pinned on a flat board, fixed in formalin, and serially sectioned at 3 mm intervals. All assessments were performed by a single gastrointestinal pathologist. The lateral and vertical resection margins were inked, and specimen orientation was maintained according to standard pathological processing protocols. Lateral margin positivity was defined as the presence of tumor cells directly involving the inked lateral resection margin, corresponding to a tumor-free horizontal margin distance of 0 mm. Lateral margin negativity was defined as the absence of tumor cells at the lateral margin, with an intervening tumor-free margin. The extent of subepithelial spread was determined by measuring the distance from the boundary of the superficial tumor to the farthest extent of tumor spread beneath the adjacent non-neoplastic epithelium.

### 2.4. Endoscopic Examination

Endoscopic images stored in the Picture Archiving and Communication System of Chungnam National University Hospital were reviewed by a single endoscopist (S.H.K.). At the time of image review, the endoscopist was blinded to the pathological lateral margin status and clinical outcomes. Lesion morphology (Paris classification), color change (redness [localized erythematous areas], discoloration [loss of normal mucosal pink hue], or mixed [combined redness and discoloration]), and anatomical location were evaluated. If subepithelial vessels were visible owing to mucosal thinning, the presence and severity of atrophic gastritis were determined by an endoscopist. If present, nodular mucosal changes were considered suggestive of intestinal metaplasia. Interobserver variability was not assessed because the image review was performed by a single experienced reviewer to ensure consistency in endoscopic feature interpretation.

### 2.5. Statistical Analysis

All statistical analyses were performed using SPSS Statistics for Windows, version 18.0 (SPSS Inc., Chicago, IL, USA). Univariate analyses were performed using the chi-square test, and multivariable logistic regression analysis was used to identify independent risk factors for LM positivity. The Mann–Whitney U test was used to compare the SE spread length between the LM positivity and LM negativity groups. Statistical significance was set at *p* < 0.05.

## 3. Results

During the study period, a total of 248 patients were included, comprising 21 patients with LM positivity and 227 patients with LM negativity. The patients’ baseline characteristics are summarized in Table 1. Most patients were men (74.6%). Most lesions were located in the antrum (58.9%) and predominantly belonged to gross type IIc (75.8%). Histologically, 41.1% and 45.2% of lesions were well and moderately differentiated, respectively. According to the Lauren classification, 89.9% of cases were intestinal type. Most lesions measured <2 cm (80.2%), and mucosal invasion was observed in 85.1% of cases. LVI was present in 6.9% of patients.

In the univariate analysis (Table 2), LM positivity was significantly associated with tumor differentiation (*p* = 0.030), Lauren classification (*p* = 0.002), lesion size (*p* = 0.007), and endoscopic color (*p* = 0.022). Presence of SE spread (*p* = 0.443), lesion location (*p* = 0.666), depth of invasion (*p* = 0.141), and LVI (*p* = 0.567) were not significantly associated with LM positivity.

Using a parsimonious binary logistic regression model (Table 3), lesion size ≥ 2 cm was significantly associated with lateral margin positivity (β = 1.478; odds ratio [OR] 4.382, 95% confidence interval [CI] 1.741–11.032; *p* = 0.002). Given the limited number of lateral margin–positive events, no additional covariates were included in the main regression model. Among patients with pathologically confirmed subepithelial spread, the mean length of subepithelial spread was greater in the lateral margin–positive group than in the lateral margin–negative group (5.80 ± 1.30 mm vs. 2.60 ± 2.36 mm). In an exploratory analysis restricted to this subgroup, subepithelial spread length ≥ 5 mm was associated with increased odds of lateral margin positivity (β = 2.713; OR 15.077, 95% CI 1.550–146.670; *p* = 0.019) (Table 3). Table 3. Binary logistic regression analyses for lateral margin positivity after endoscopic submucosal dissection.

The exploratory analysis was restricted to patients with pathologically confirmed subepithelial spread. Given the limited number of LM-positive events, parsimonious models were used and the results are interpreted as associations.

## 4. Discussion

ESD can effectively treat EGC with a low risk of lymph node metastasis, offering overall survival outcomes comparable to those of surgical resection. However, because ESD preserves the stomach, it is associated with a higher local recurrence rate than surgery [5]. A Korean study identified several risk factors for local recurrence after ESD, including large tumor size, incomplete histologic resection, undifferentiated histology, presence of scar ring, and absence of mucosal redness [6]. Incomplete resection refers to the presence of residual cancer cells at the resection margin and can be categorized as either LM or vertical margin involvement. Vertical margin positivity often suggests that surgical resection should be considered from the outset rather than ESD. Therefore, minimizing LM positivity after ESD is crucial to reduce local recurrence and avoid unnecessary additional treatments.

Previous studies on LM positivity reported several risk factors, including larger tumor size, location in the upper third of the stomach, undifferentiated histology, lesions beyond the absolute indication criteria, depressed-type morphology, and submucosal invasion [1,7,8]. The underlying causes of LM positivity can be categorized into lesions that are technically challenging to resect (owing to size or location), those in which endoscopists cannot clearly delineate tumor margins (undifferentiated histology), and those that fall outside the standard indications for ESD. Although these studies did not specifically address the endoscopist’s experience, the fact that most ESD procedures are performed by experienced operators raises the question of whether factors beyond technical difficulty alone contribute to LM positivity. A previous study from Korea reported that misjudgment of the lesion boundary during ESD was a major cause of LM positivity [2]. Although even expert endoscopists may make mistakes influenced by factors such as fatigue or workload, this raises the question of whether LM positivity is solely due to human error or if lesions have intrinsic characteristics that make accurate boundary identification inherently difficult. Accordingly, this study aimed to clarify whether SE spread could serve as an intrinsic lesion-related factor leading to LM positivity beyond simple endoscopist-related technical limitations.

Although LM positivity and residual disease are not synonymous, clinical overlap exists. A Korean study evaluated residual disease after ESD, defining it as local recurrence near the previous resection site despite a pathologically complete resection [9]. This study identified a safety margin of ≤3 mm, signet ring cell carcinoma (SRC), and severe intestinal metaplasia (IM) as significant risk factors. In cases of severe IM, the development of new lesions near the resection site may not be surprising even after complete removal of the original lesion, as substantial cellular damage may have already accumulated in the surrounding mucosa. A narrow safety margin, similar to severe IM, may serve as a risk factor for residual disease, as it may reflect the incomplete removal of mucosa that has undergone substantial cellular damage [10]. SRC contributes to residual disease by frequently exhibiting SE spread beneath normal-appearing mucosa, a phenomenon particularly characteristic of infiltrative spreading [11]. If SE spread is present, the endoscopist’s perceived extent of the lesion may differ from its true pathologic extent, leading to LM positivity. Previous studies investigating the therapeutic outcomes of endoscopic resection for undifferentiated-type gastric cancer reported that after ESD, LM positivity is more commonly observed in SRC, whereas deep margin positivity occurs more frequently in other forms of undifferentiated cancer [12]. These observations prompted us to question whether factors such as SE spread, which can hinder the accurate endoscopic delineation of tumor margins, are crucial in LM positivity. Therefore, this study was designed to investigate that possibility.

We previously reported that SE spread in SRC can extend up to 6 mm [13]. SE spread can occur frequently in other undifferentiated cancers and mixed-type cancers; previous studies reported that SE spread can extend up to 17 mm and 10 mm in mixed-type and other undifferentiated cancers, respectively [4]. Considering that endoscopists generally mark 3–5 mm beyond the tumor boundary during ESD, SE spread is a plausible mechanism underlying LM positivity; however, this association remains underexplored.

In our study, the presence of SE spread was not significantly associated with LM positivity. This may be attributable to the limited number of undifferentiated cancers in our cohort, in which SE spread is known to occur more frequently. Only 28.6% (6/21) and 9.3% (21/227) of the LM-positive and control groups, respectively, had an undifferentiated histology, suggesting that the sample size was insufficient to adequately evaluate this association. This likely reflects the fact that ESD is predominantly performed for lesions initially diagnosed as differentiated cancers. Although SE spread can occur in differentiated-type cancers, our previous study showed that its prevalence was significantly lower in these lesions (19.4%) compared with undifferentiated cancers (76.1%) [4]. However, this study demonstrated that the mean length of SE spread was significantly greater in the LM-positive group than in the control group. Among patients with SE spread, the mean extent was significantly longer in the LM-positive group (5.80 ± 1.30 mm) than in the control group (2.60 ± 2.36 mm) (*p* = 0.004, independent *t*-test). Logistic regression analysis revealed a statistically significant difference in the mean length of SE spread between the two groups. These findings suggest that if endoscopists are unaware of the presence of SE spread and perform conventional marking, LM positivity may occur. Thus, LM positivity cannot be fully explained by operator-dependent factors alone; certain biological growth patterns, such as SE spread, may inherently limit the accuracy of endoscopic boundary delineation. Moreover, the mere presence of SE spread may be insufficient to influence margin status; rather, the extent of SE spread appears to be the determining factor. This suggests that SE spread becomes clinically relevant only if it exceeds the typical resection margin width used during ESD. Additionally, the differentiation status, lesion size, and lesion color change were statistically associated with LM positivity, consistent with previous studies.

Because ESD is conventionally performed by placing marking dots approximately 3–5 mm outside the visible tumor margin, followed by circumferential incision outside the markings, we used a 5 mm cutoff to evaluate whether the extent of SE spread influenced LM positivity. Analyzing whether SE spread ≥5 mm affected LM positivity, we found that 5 mm is a clinically meaningful cutoff, and logistic regression analysis demonstrated that the presence of SE spread ≥5 mm was associated with a 15-fold increase in the odds of LM positivity (Table 3). Therefore, the conventional margin setting used during ESD may require expansion, particularly in lesions with a higher likelihood of SE spread, such as undifferentiated cancers or larger tumors, for which a wider resection may be necessary to prevent LM positivity. For lesions with undifferentiated histology or mixed color change—features associated with SE spread—wider resection margins may be warranted, although the tumor boundary appears well demarcated endoscopically.

This study has several strengths. First, it directly evaluated the relationship between the pathologic extent of SE spread and LM positivity, providing mechanistic insights beyond operator-dependent factors. Second, by quantitatively correlating the SE spread length with the conventional ESD 3–5 mm marking margin, the findings offer clinically meaningful guidance regarding margin selection. Third, SE spread ≥5 mm was identified as an independent predictor of LM positivity, demonstrating a strong and clinically relevant effect size. Finally, all procedures were performed by highly experienced endoscopists, and the pathologic assessment was standardized to minimize variability and enhance reliability. These findings may help standardize the marking strategy during ESD and potentially reduce the incidence of LM positivity. Securing an adequate safety margin to achieve curative resection is a key component of effective treatment for early gastric cancer. A Korean study demonstrated that a narrow safety margin of ≤1 mm was associated with an increased risk of local recurrence [14]. Although LM positivity is not directly associated with worse survival outcomes, its association with increased local recurrence underscores the importance of securing an adequate resection margin [15], as this may reduce the need for additional endoscopic or surgical interventions. Current guidelines from multiple countries recommend additional endoscopic resection or surgical treatment in cases of lateral margin positivity; therefore, securing an adequate resection margin may help reduce unnecessary additional procedures or surgery [16,17,18].

### Limitation and Future Directions

However, this study has several limitations. This was a single-center retrospective study, and the number of LM-positive cases, particularly those with undifferentiated histology, was relatively small, which may have limited the statistical power. Retrospective designs have inherent methodological limitations, including reliance on existing records not originally intended for research, potential for missing or incomplete data, selection bias, and an inability to establish causal relationships, which may affect the interpretation of findings [19]. Because lateral margin positivity is a relatively rare event, LM-positive cases were accumulated over multiple years to secure an adequate sample size, whereas the control group was restricted to a single year to allow consistent endoscopic and pathological reassessment. All procedures were performed by endoscopists who had already surpassed the learning curve for gastric ESD, and procedural techniques were largely standardized during the study period, which may have mitigated the impact of temporal variability. Although a standard marking distance of approximately 3–5 mm was routinely used, minor operator-dependent variability in marking technique may have existed, which could not be fully quantified in this retrospective analysis. SE spread was measured pathologically using 3 mm tissue sections, which may introduce measurement variability. Previous studies have reported that variations in section interval may influence the detection rate of microscopic invasion or margin involvement. In particular, section intervals greater than 2 mm have been associated with a significantly reduced detection rate of minimal submucosal invasion or lymphovascular invasion [20]. Lastly, we were unable to evaluate whether the prospective application of wider resection margins would effectively reduce LM positivity. Despite these limitations, the present study provides quantitative pathological evidence supporting a mechanistic role of subepithelial spread in lateral margin positivity. Future multicenter, prospective studies with larger sample sizes are warranted to validate these findings and to determine whether specific pre-ESD endoscopic or clinicopathologic features can reliably predict the presence or extent of subepithelial spread. The effect estimate for SE spread ≥5 mm was imprecise, reflecting the small sample size, particularly in the LM-positive subgroup. Such studies may help refine margin-setting strategies and optimize patient selection for endoscopic submucosal dissection.

Overall, our findings suggest that SE spread, particularly if its extent is >5 mm, may represent a previously under-recognized mechanism of LM positivity; awareness of this factor may help refine marking strategies and reduce incomplete lateral resections.

## 5. Conclusions

In conclusion, the extent of subepithelial spread was associated with lateral margin positivity after endoscopic submucosal dissection for early gastric cancer. Although the presence of subepithelial spread alone was not sufficient to determine margin status, a spread length of five millimeters or more showed a strong association with lateral margin positivity. Importantly, subepithelial spread was evaluated pathologically after resection and is not directly assessable during pre-ESD endoscopic evaluation. Therefore, these findings should not be interpreted as establishing definitive pre-ESD predictors of subepithelial spread. Rather, they provide mechanistic insight into why lateral margin positivity may occur despite apparently adequate endoscopic delineation, highlighting the role of intrinsic tumor growth patterns beyond technical factors. Further prospective studies are warranted to validate these observations.

## Figures and Tables

**Table 1:** Demographics.

Characteristics	Number of Patients (%)
Gender (M:F)	185:63
Location	
Cardia	1 (0.4)
Body	61 (24.6)
Angle	40 (16.1)
Antrum	146 (58.9)
Gross type	
I	3 (1.2)
IIa	30 (12.1)
IIb	27 (10.9)
IIc	188 (75.8)
Differentiation	
Well	102 (41.1)
Moderate	112 (45.2)
Moderate to poor	7 (2.8)
Poor	27 (10.9)
Lauren	
Intestinal	223 (89.9)
Diffuse	21 (8.5)
Mixed	4 (1.6)
Size	
<1 cm	108 (43.5)
1 cm≤ <2 cm	91 (36.7)
2 cm≤ <3 cm	35 (14.1)
3 cm≤	14 (5.6)
Positive lateral margin (Y:N)	21:227
Depth of Invasion (M:SM)	211:37
Positive LVI (Y:N)	17:231

**Table 2:** Univariate analysis for positive lateral margin after ESD.

	Margin (+)	Margin (−)	*p* Value
Presence of SE			0.443
(+)	5	63	
(−)	16	164	
Location			0.666
Cardia	0	1	
Body	6	55	
Angle	5	35	
Antrum	10	136	
Differentiation			0.030
Well	5	97	
Moderate	10	102	
Moderate to poor	0	7	
Poor	6	21	
Lauren			0.002
Intestinal	15	208	
Diffuse	4	17	
mixed	2	2	
Size			0.007
<1 cm	4	104	
1 cm≤ <2 cm	7	84	
2 cm≤ <3 cm	7	28	
3 cm≤	3	11	
SM invasion			0.141
M cancer	16	195	
SM cancer	5	32	
LVI			0.567
(+)	1	16	
(−)	20	211	
Endoscopic color			0.022
Redness	2	91	
Mixed	11	80	
Discolored	8	56	

**Table 3:** Binary logistic regression analyses for lateral margin positivity after endoscopic submucosal dissection.

Analysis	Variables	β-Coefficient	OR	95% CI	*p* Value
Main analysis (overall cohort)	Lesion size ≥ 2 cm (vs. <2 cm)	1.478	4.382	1.741–11.032	0.002
Exploratory analysis (SE spread–positive subgroup)	SE spread length ≥ 5 mm (vs. <5 mm)	2.713	15.077	1.550–146.670	0.019

## Data Availability

The data presented in this study are not publicly available due to privacy and ethical restrictions related to patient information. De-identified data may be made available from the corresponding author upon reasonable request and with appropriate institutional approval.
